# Metal electrodes transfer using polycarbonate for the fabrication of molybdenum disulfide semiconductor devices

**DOI:** 10.1186/s11671-026-04877-z

**Published:** 2026-08-26

**Authors:** Chih-Hao Chiang, Ruo-Yao Wang, Jing-Ting Chou, Yun-Ping Chiu, Che-Wei Chu, Yueh-Wei Chen, Guan-Ting Chen, Zi-Rui Su, Chi Chen, Chen-Fang Kang, Jhong-Ren Huang, Meng-Lin Tsai

**Affiliations:** 1 Department of Materials Science and Engineering, National Taiwan University of Science and Technology, Taipei City, 106335 Taiwan; 2 Department of Mechanical Engineering, National Taiwan University of Science and Technology, Taipei City, 106335 Taiwan; 3 Department of Electronic Engineering, Chung Yuan Christian University, Taoyuan City, 320314 Taiwan; 4 Research Center for Applied Sciences, Academia Sinica, Taipei City, 115201 Taiwan

**Keywords:** Polycarbonate, Transition metal dichalcogenides, Photodetectors, Field effect transistors, Metal electrode transfer

## Abstract

Fabricating high-performance 2D nanosheet-based semiconductor devices faces challenges due to the structural damage caused by traditional metal electrode deposition processes. This study introduces a novel polycarbonate (PC) assisted metal electrode transfer technique that effectively preserves the structural and electronic properties of MoS_2_ nanosheets. Using the proposed method, metal electrodes are successfully transferred onto MoS_2_ nanosheets via the shadow mask process (20 μm channel) and photolithography (8 μm and 3 μm channels) for photodetector and field-effect transistor (FET) fabrication, respectively. Photodetectors fabricated with transferred electrodes exhibit stable current-voltage characteristics, with the shadow mask-based photodetector achieving a maximum photoresponsivity of 0.73 µA/W under a 450 nm laser at 10 V bias. The photolithography-based photodetector demonstrates higher photoresponsivity of 3.62 mA/W due to enhanced light absorption. Additionally, the FETs fabricated using the PC transfer process show NMOS behavior with electron mobility values of up to 0.17 cm^2^/V s during reverse sweeps. PC covered FETs further improve performance under ambient conditions, achieving electron mobility up to 0.517 cm^2^/V s and stable switching current ratios. These findings underscore the PC assisted electrode transfer method’s advantages in maintaining material integrity, achieving stable device performance, and offering scalability for large-scale applications.

**Supplementary Information:**

The online version contains supplementary material available at 10.1186/s11671-026-04877-z.

## Introduction

To avoid reaching physical limits and extend Moore’s Law, finding next-generation alternative materials has become an urgent issue. Two-dimensional materials such as graphene, hexagonal boron nitride (hBN), and transition metal dichalcogenides (TMDs) have garnered significant attention and are considered potential solutions [1–3]. Among these, TMDs are particularly promising as they meet the required specifications. Their bandgap falls within the semiconductor range, and they exhibit tunable bandgap properties, high carrier mobility, flexibility, and high transparency, making them valuable for applications in electronics, optoelectronics, and photonics [4–6]. These unique properties have positioned TMDs as key candidates for next-generation electronic and optoelectronic devices [7, 8].

However, research and development of TMDs face two significant challenges. The first challenge involves achieving the growth of large-area, high-quality, and layer-controllable nanosheets and thin films. This is crucial for ensuring the stability and performance of semiconductor devices [9–11]. The second challenge lies in developing fabrication methods that preserve the material’s structural and functional integrity during device assembly. Addressing these challenges has become a global research focus, with numerous studies exploring novel approaches to overcome the limitations of existing techniques [12, 13]. Traditional electrode fabrication methods for TMD devices typically involve direct metal deposition onto the TMD layer using techniques such as evaporation or sputtering. However, these high-energy deposition processes can induce significant damage to the TMD layer. Transmission electron microscopy (TEM) cross-sectional images reveal defects caused by metal particle impacts, metal diffusion into the TMD layer, and stress-induced lattice distortion. These damages often lead to atomic disorder and the formation of amorphous layers, compromising the device’s functionality. Additionally, after electrode removal, the TMD layer beneath the metal electrode is often found to be completely destroyed, rendering the device inoperable [14–16]. These limitations underscore the urgent need for alternative electrode fabrication methods to preserve the TMD layer’s integrity and ensure device reliability [17, 18].

Metal electrode transfer techniques have been developed to address these issues. These methods typically involve depositing metal electrodes onto a substrate, adhering them to a polymer film, and subsequently transferring them onto the TMD layer. By avoiding high-energy impacts, these techniques maintain the structural integrity of the TMD material, enabling the fabrication of functional devices. However, many existing transfer methods require complex substrate pretreatment, the use of multiple polymers, and additional steps such as chemical etching or dissolution, which increase both the cost and complexity of the process [19, 20]. For example, techniques employing hexamethyldisilazane (HDMS)-functionalized substrates or polyvinyl alcohol (PVA) films often involve multiple coating and removal steps and rely on chemical solutions for layer separation, making these methods labor-intensive and time-consuming [21, 22].

This study proposes a novel electrode transfer method utilizing polycarbonate (PC) as the transfer polymer. Unlike existing techniques, this method eliminates the need for substrate pretreatment and chemical etching. Metal electrodes are deposited onto Si or SiO_2_/Si substrates, coated with a PC layer, and subsequently peeled off and transferred to the TMD layer. Using PC as a single polymer material significantly simplifies the process, requiring fewer steps and reducing fabrication costs. Furthermore, the process is faster and easier to implement than conventional methods [23, 24]. The advantages of the proposed method are evident when compared to existing techniques. Its simplified workflow and cost-effectiveness make it particularly suitable for large-scale applications in TMD device fabrication. Furthermore, this study evaluates the performance of the transferred electrodes by fabricating photodetectors (PDs) and field-effect transistors (FETs). The results demonstrate that the proposed method preserves the integrity of the TMD layer while ensuring reliable device performance, offering a practical and scalable solution for future advancements in TMD-based technologies [25].

## Methods

### Growth of molybdenum disulfide nanosheets and fabrication of device electrodes

Monolayer MoS_2_ nanosheets were synthesized using low-pressure chemical vapor deposition (CVD) on single-side polished sapphire substrates. Substrates were cut into 1.5 cm × 2.0 cm pieces, ultrasonically cleaned in acetone, IPA, and DI water for 15 min, and treated with ultraviolet ozone (UVO) for 20 min to ensure cleanliness. Pre-treated substrates, sulfur powder (400 mg), molybdenum trioxide (200 mg), and polished sapphire substrates were placed in tungsten boats and loaded into a quartz tube. The tube was evacuated to 20 mtorr, pressurized to 30 torr with argon (80 sccm). Furnace 1 and furnace 2 were heated to 160 °C (17 °C min^− 1^) and 750 °C (24 °C min^− 1^), respectively. After a 40-minute process, the system was cooled to room temperature, yielding MoS_2_ nanosheets (Fig S1 and S3). The PC solution was prepared by sonicating PC pellets and cyclopentanone (1.2 g:10 g) for 1 h to ensure dissolution (Fig. S4). Metal electrodes were fabricated via two processes. In the shadow mask method, pre-treated silicon wafers (1 cm × 1 cm) were masked and sputtered with gold at 40 mA for 200 s to form interdigitated electrodes (20 μm channel length, Fig. S5). In the photolithography method, silicon wafers were cleaned and coated with 100 nm SiO_2_, spin-coated with positive photoresist (1.2 μm), soft-baked, aligned with photomasks, exposed, and developed. Gold electrodes were deposited by electron beam evaporation, and residual photoresist was removed. This process produced electrodes with 8 μm channel lengths for photodetectors and 3 μm for FETs (Fig. S6).

### Transfer of MoS_2_ nanosheets

**Fig. 1:**
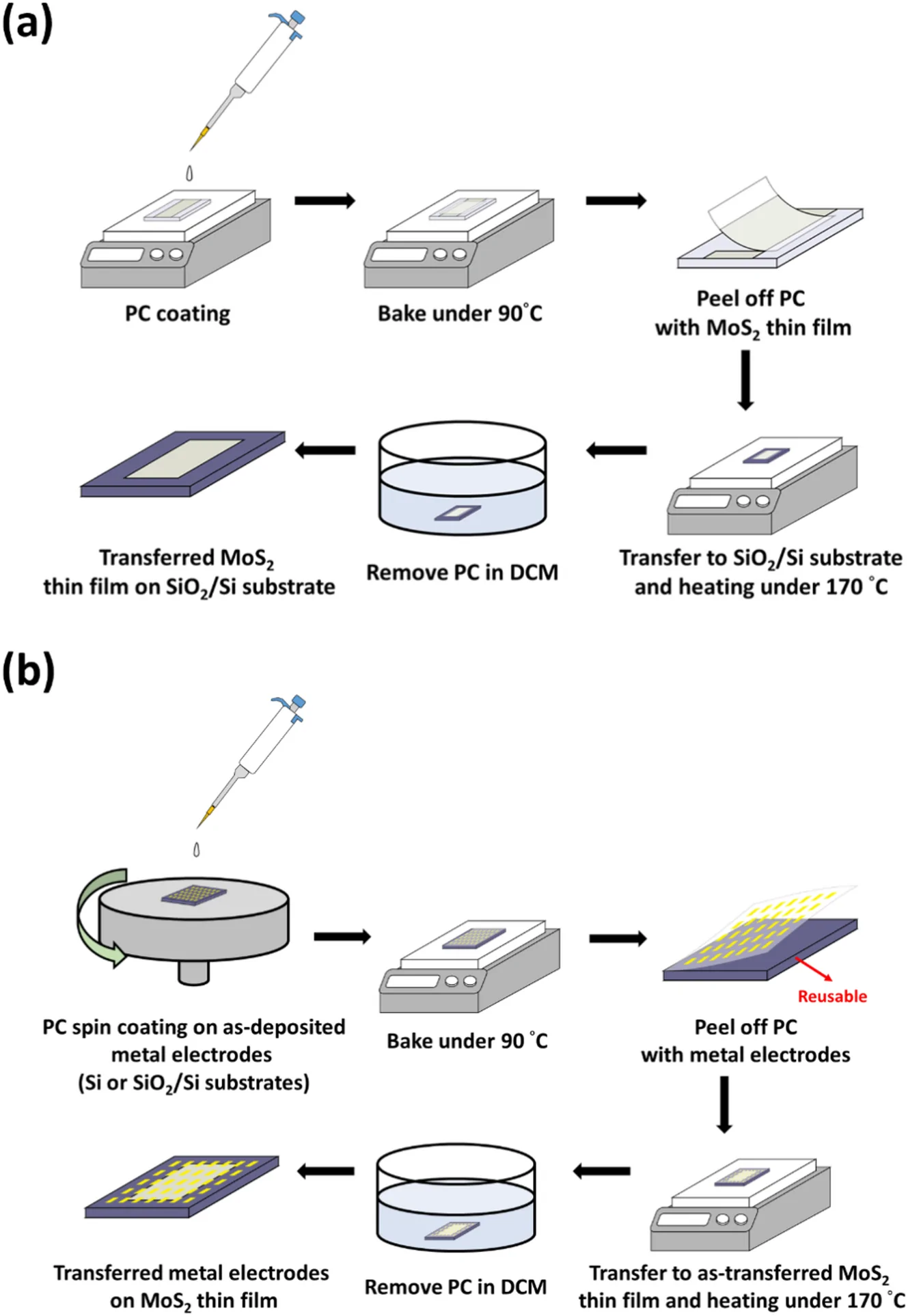
Schematic illustration of the **a** MoS_2_ nanosheet transfer process and **b** metal electrode transfer process for the field-effect transistor

MoS_2_ nanosheets were transferred to SiO_2_/Si substrates using a PC transfer process. PC solution (20 µL) was evenly spread on the sapphire substrate and soft-baked at 90 °C for 20 min (Fig. 1a). The solidified film and MoS_2_ nanosheet were lifted with tweezers, placed on SiO_2_/Si substrates, heated at 170 °C for 30 min, and cooled. Dichloromethane was used to dissolve the PC, and a nitrogen gun was used to remove residual solvents. For metal electrodes, 80 µL of PC solution was applied to substrates with electrodes, spin-coated at 1000 rpm for 10 s, and soft-baked at 90 °C for 20 min (Fig. 1b). The solidified film and electrodes were removed with tweezers, transferred to MoS_2_ nanosheets, heated at 170 °C for 30 min, and dissolved in dichloromethane to complete the clean transfer process.

### Photodetector and field-effect transistor fabrication and characterizations

Metal electrode transfer was employed to fabricate photodetectors and FETs. Photodetector electrodes were fabricated using the shadow mask method (20 μm channel) or photolithography (8 μm channel), while FET electrodes (3 μm channel) were prepared exclusively through photolithography. The photodetectors feature a top-contact structure (Fig. S7). PC solution (80 µL) was spin-coated on silicon substrates at 1000 rpm for 10 s, soft-baked at 90 °C for 20 min, and removed along with the metal electrodes using tweezers. The assembly was placed on MoS_2_ nanosheets, heated at 170 °C for 30 min, and cooled. Dichloromethane dissolved the PC, completing the fabrication process (Fig. S8). Photodetector performance was characterized by measuring current-voltage and current-time characteristics under laser illumination (430 nm, 520 nm, 650 nm) with varying power levels. Results were used to calculate the photoresponse rate, on/off current ratio, and response time. FETs were fabricated with a bottom-gate top-contact structure (Fig. S9). Electronic properties, including drain current–gate voltage and the square root of drain current–gate voltage curves under various drain voltages, were measured (See Supplementary material for more details).

## Results and discussion

### Material analysis of MoS_2_ nanosheets

**Fig. 2:**
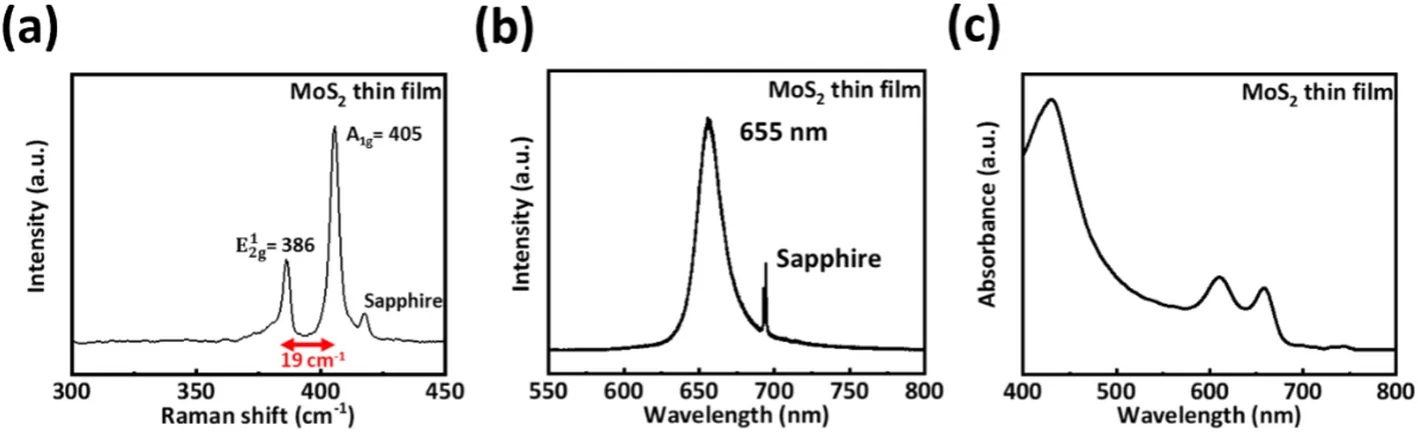
**a** Raman, **b** PL, and**c** UV-vis-NIR absorbance spectra of MoS_2_ nanosheets

Monolayer MoS_2_ nanosheets were synthesized using low-pressure CVD on single-side polished sapphire substrates. As shown in Fig. S3, MoS_2_ microflakes, continuous films, and multilayer films can be successfully grown in regions I, II, and III, respectively. The average lateral size of the continuous-film region used for transfer (region II) is approximately 1 cm × 1 cm. Furthermore, based on the optical microscopic images shown in Fig. S10, the transferred MoS_2_ film retains approximately 95% coverage compared to the original as-grown continuous film, indicating that the transfer process preserves the film continuity over a large area. To confirm the successful growth of MoS_2_ nanosheets and to make a preliminary assessment of their number of layers, Raman spectroscopy and photoluminescence (PL) spectroscopy measurements were conducted, as shown in Fig. 2a and b. The Raman spectrum reveals characteristic peaks at 386 cm^− 1^, 405 cm^− 1^, and 417 cm^− 1^, which correspond to the in-plane vibration ($\:{\mathrm{E}}_{\mathrm{2g}}^{\mathrm{1}}$) and out-of-plane vibration (A_1g_) modes of MoS_2_, as well as signals from the sapphire substrate. This confirms that the grown sample is indeed MoS_2_. Additionally, calculating the difference in Raman shifts between the two characteristic peaks yields Δω of approximately 19 cm^− 1^ indicates that the MoS_2_ nanosheets are likely monolayers (Δω < 20 cm^− 1^). The PL spectrum shows emission peaks at 655 nm and 694 nm, corresponding to the A-exciton emission from MoS_2_ and the emission signal from the sapphire substrate, respectively. The A-exciton emission signal is attributed to direct bandgap emission, allowing for the estimation of the bandgap energy to be approximately 1.89 eV, which aligns with the expected range for monolayer MoS_2_ (1.8 eV–1.9 eV).

To confirm the wavelength absorption range of the MoS_2_ nanosheets, the absorption spectrum was measured using a UV-visible-near infrared spectrometer (UV-vis-NIR). As depicted in Fig. 2c, the absorption spectrum displays peaks at 658 nm, 611 nm, and 430 nm, corresponding to A-exciton absorption, B-exciton absorption, and C-exciton absorption, respectively. The calculated optical bandgap is approximately 1.84 eV from the Tauc plot shown in Fig. S11. The generation of the A-exciton and B-exciton absorption peaks is attributed to valence band splitting due to spin-orbit coupling and carrier transitions between the minimum of the conduction band at the K point in the Brillouin zone. The C-exciton absorption peak is attributed to carrier transitions between the deep valence and conduction bands at the M point in the Brillouin zone [8, 9].

**Fig. 3:**
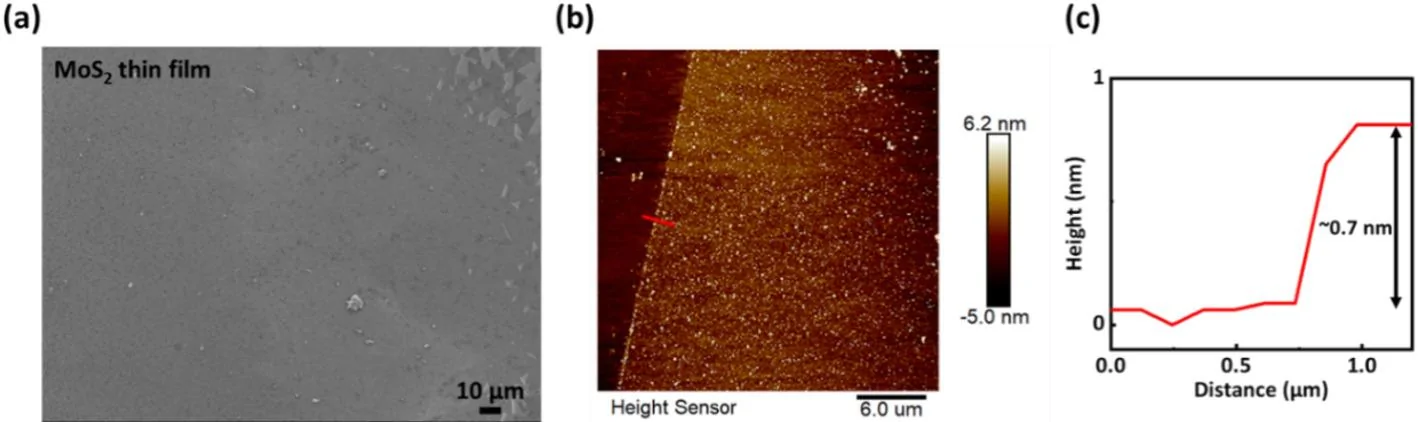
**a** SEM image (500×), **b** AFM image, and **c** AFM height profile curve of MoS_2_ nanosheets

To confirm the surface morphology and thickness of MoS_2_, scanning electron microscopy (SEM) and atomic force microscopy (AFM) measurements were conducted. SEM image of the MoS_2_ nanosheets is shown in Fig. 3a, where single-crystal MoS_2_ triangular flakes can be observed in the upper right corner. This demonstrates that the synthesized MoS_2_ nanosheets exhibit excellent poly-crystallinity. When the substrate is positioned closer to the precursors during the CVD process, a higher gas concentration promotes the growth of continuous polycrystalline films. Conversely, as the substrate moves further away from the precursors, the gas concentration decreases, forming smaller triangular flakes from continuous polycrystalline nanosheets. AFM image of the MoS_2_ nanosheets is shown in Fig. 3b, with the dark region on the left representing the sapphire substrate and the right side representing the MoS_2_ nanosheets. The corresponding height profile curve obtained from the red line in Fig. 3b is shown in Fig. 3c, indicating that the thickness of the MoS_2_ nanosheets is approximately 0.7 nm, further confirming that the MoS_2_ nanosheets grown in this study are monolayers.

### Metal electrode transfer

The metal-masked photodetector was selected as the comparing device to compare the effect of different fabrication methods, namely the electrode transfer process and electrode deposition process, on the MoS_2_ nanosheets. The MoS_2_ nanosheets grown via the same CVD process were subjected to electrode transfer and deposition processes, followed by the removal of the electrodes to observe any changes in the MoS_2_ nanosheets. Optical microscope (OM) images with an inset of actual photos before (left image) and after (right image) electrode removal for the two electrode fabrication processes are presented in Fig. S12a and b. Raman spectroscopy (Fig. S13a) and PL spectra (Fig. S13b) were utilized to evaluate changes in the MoS_2_ nanosheets before and after electrode transfer and deposition processes [26, 27]. After the electrode deposition process, the Raman spectrum of the MoS_2_ nanosheets revealed a reduction in the intensity of characteristic peaks and the appearance of peaks corresponding to MoO_x_S_y_ (Fig. S13a). Accompanying this, the OM image in Fig. S12b shows damage to the channel region of the MoS_2_ nanosheets, indicating an undesired interaction between the MoS₂ nanosheets and electrodes, leading to a deterioration of the MoS_2_ characteristics. In contrast, the Raman spectrum of the MoS_2_ nanosheets after the electrode transfer process displayed no significant changes. Similarly, the PL spectra in Fig. S13b show that the intensity of the MoS_2_ characteristic peaks significantly decreased or disappeared after the electrode deposition process. Conversely, the distinct peaks remained prominent after the electrode transfer process. These findings suggest that the high-energy conditions required for the electrode deposition process, which involve ionizing the metal target and depositing it onto the MoS_2_ nanosheets, result in structural damage and the formation of defects. This process likely causes metal diffusion into the MoS_2_ nanosheets and the formation of MoO_x_S_y_ [28].

Further analysis using AFM surface morphology images in Fig. S11 was performed to assess the impact of the transfer process on electrode thickness and surface roughness. The root-mean-square roughness (R_q_) of the electrodes before and after the transfer, as shown in Fig. S14a and c, was found to be 14.2 nm and 16.8 nm, respectively. This difference is not significant concerning the overall electrode thickness. The AFM height profile curves in Fig. S14b and d indicate that the thickness of the electrodes before and after the transfer is consistently 100 nm, demonstrating that the transfer process does not significantly affect the electrode thickness.

To evaluate changes in the overall electrode and electrode channel lengths during the shadow mask electrode transfer process, OM and photographic images were obtained at each step, as shown in Fig. S15. Figure S15a illustrates the low magnification OM and photographic images of the electrode before transfer, while Fig. S15b shows the high magnification OM image of the electrode channel before transfer, with a channel length of approximately 20 μm. Figure S15c presents the original substrate’s OM and photographic images after the electrode transfer, where the electrodes have been entirely removed. Figure S15d depicts the OM and photographic images of the electrode on the PC film, confirming that the electrodes have been successfully detached. Figure S15e highlights the OM image of the electrode on the PC film at the channel region, showing that the electrode channel length remains at approximately 20 μm. Figure S15f shows the OM and photographic images of the transferred electrode on the MoS_2_ nanosheets, indicating that the electrode has been wholly transferred, although some PC residue is still visible. Finally, Fig. S15g illustrates the OM image of the electrode channel after transfer, maintaining a channel length of about 20 μm. These findings confirm that using PC film for metal electrode transfer preserves the electrode’s integrity and ensures the channel length remains unaffected.

**Fig. 4:**
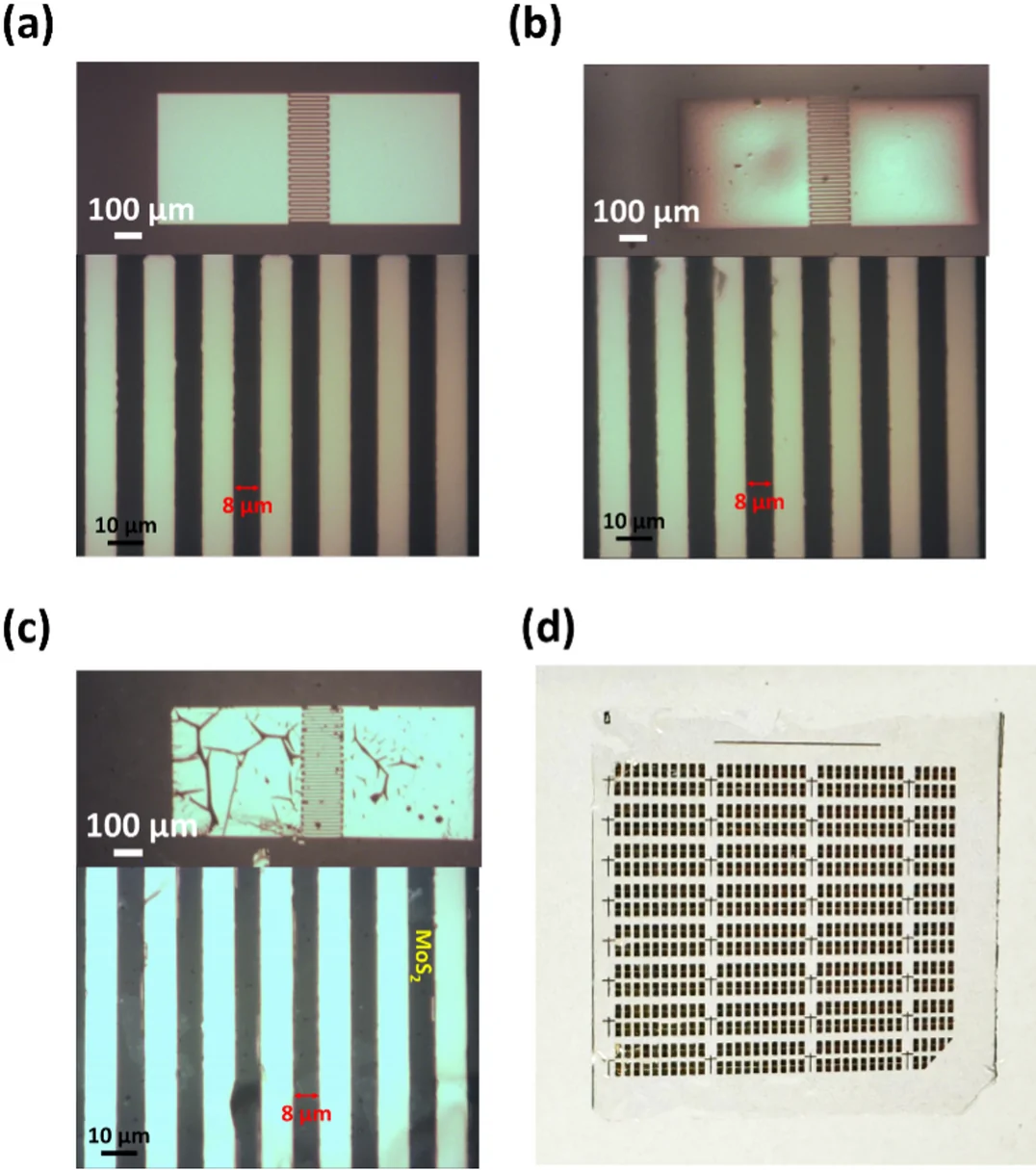
**a** Photolithographic electrodes and channel OM images before transfer, **b** on the PC film, and **c** after transfer. **d** Photographic image of the electrodes on the PC film

From low- and high-magnification OM images (Fig. 4a and c) of the electrode transfer process deposited by photolithography, it is shown that the metal electrodes can be removed entirely by the PC film and transferred to the MoS_2_ nanosheets, although some PC residue remains [29]. The high-magnification OM images confirm that the positions of the interdigitated electrodes were fully transferred, with the channel length remaining approximately 8 μm without deformation. Figure 4d shows a photo of the interdigitated electrodes on the PC film, which is 1/4 of a 4-inch photomask size (approximately 3 cm × 3 cm). In this area, the electrodes were removed entirely and transferred.

**Fig. 5:**
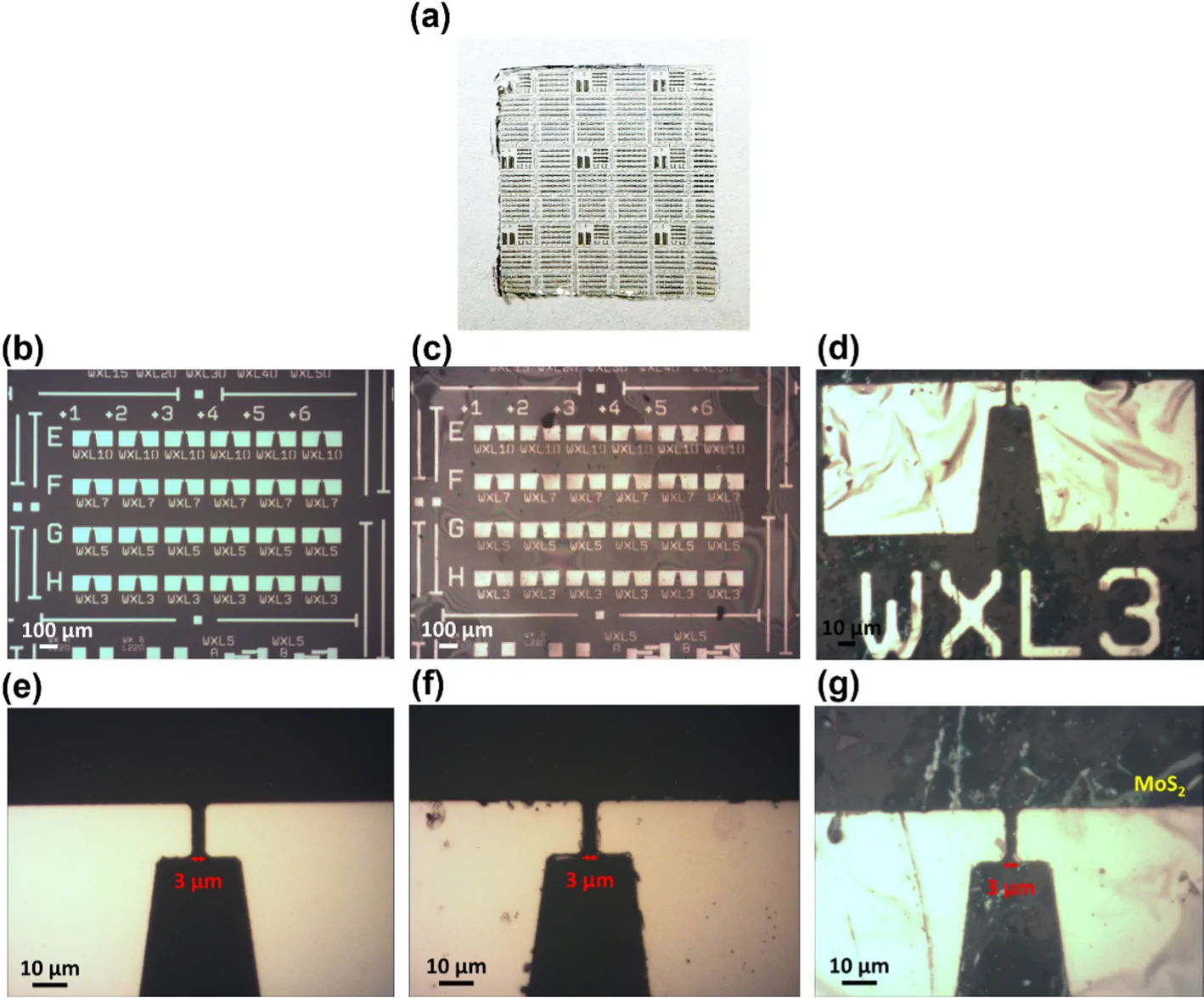
**a** Photographic image of FET electrodes on the PC film. Low-magnification OM images of FET electrodes **b** before transfer, **c** on the PC film, and **d** after transfer. High-magnification OM images of the channel **e** before transfer, **f** on the PC film, and **g** after transfer

Figure 5a depicts the photographic image of FET electrodes on the PC film, which can also be entirely removed from the substrate. Figure 5b and d present OM images at different stages of the FET electrode transfer process. This photomask contains several FET electrodes with varying channel lengths. The analysis focuses on the most petite channel length (3 μm). It is evident that regardless of the channel length, the PC film successfully removed and transferred the FET electrodes to the MoS_2_ nanosheets, although some PC residue remains. Figure 5e and g display the OM images of the channel regions during the transfer process, showing that the channel length remains approximately 3 μm without significant changes.

### MoS_2_ nanosheet photodetector

To analyze the interface properties between the MoS_2_ nanosheet and the electrodes after the electrode transfer and the performance of the photodetector, this study measures photodetectors fabricated using the shadow mask and photolithographic electrode processes and discusses the measurement results. Figure S16a–c show the I-V characteristic curves of the photodetector using the shadow mask electrode process under laser wavelengths of 450 nm, 520 nm, and 650 nm, respectively, with varying laser powers. The voltage and current exhibit a linear relationship, indicating that the interface between the shadow mask electrode and the MoS_2_ nanosheet is ohmic contact. Figure S16d–f depict the photodetector’s photoresponsivity under different laser powers and biases at the same wavelengths. Under the low power of the 450 nm laser at 10 V, the maximum photoresponsivity is approximately 0.73 µA W^−1^; for the 520 nm laser at 10 V, it is around 0.22 µA W^−1^; and for the 650 nm laser at 10 V, it is approximately 0.33 µA W^−1^.

Figure S17a–c present the I-t curves of the photodetector under the three laser wavelengths at a constant bias of 10 V, demonstrating stable light and dark current performance over multiple cycles within a 30-second interval. Figure S17d–f illustrate the I-t curves for single switches. The results indicate that the switching current ratio for the photodetector at 10 V is approximately 102 for the 450 nm and 650 nm lasers, while the switching current ratio for the 520 nm laser is about 10. Figure S18a–f displays the response times of the photodetector under 10 V for the 450 nm, 520 nm, and 650 nm lasers (defined by the interval between 10 and 90% of the photocurrent). Regardless of the laser wavelength, the rise time of the photodetector is approximately 145 ms, and the fall time is about 134 ms.

**Fig. 6:**
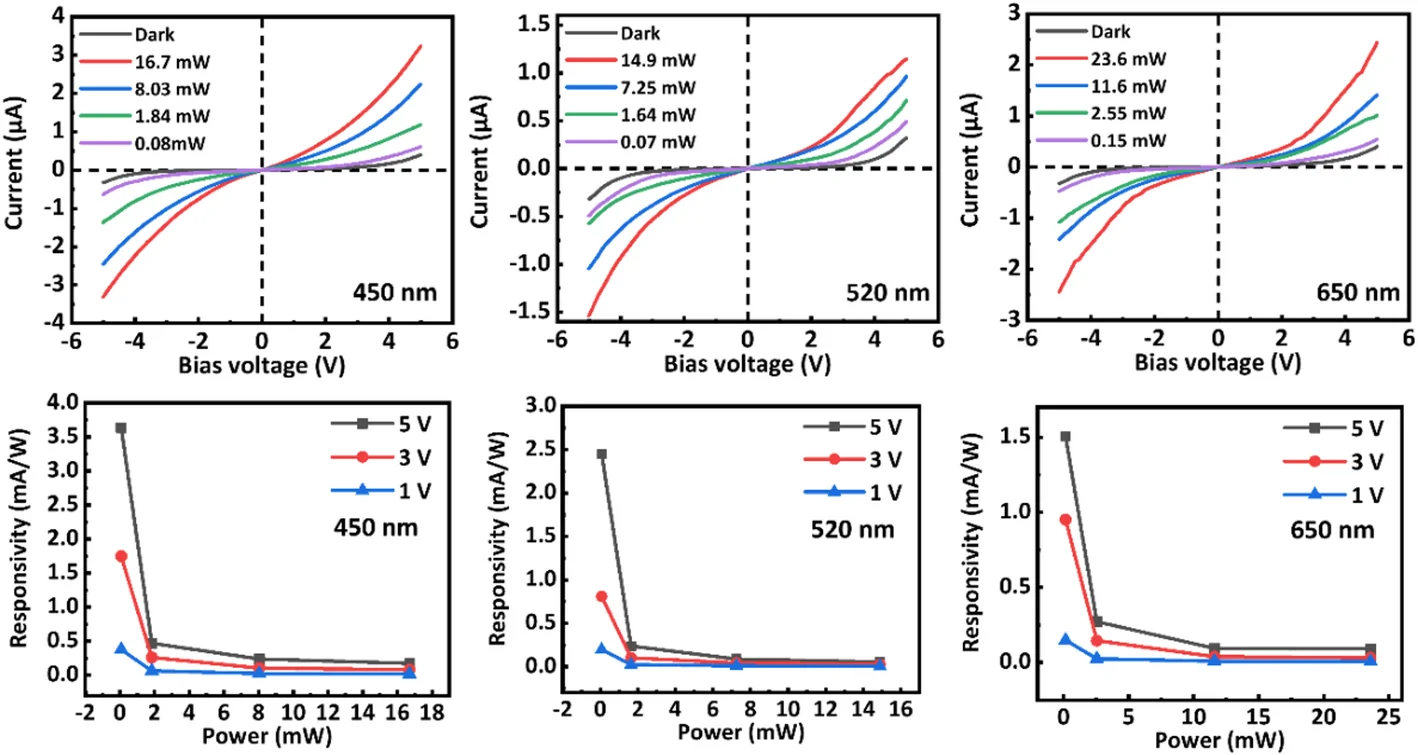
**a** I-V characteristic curves of the photodetector with photolithographic electrode process under 450 nm, **b** 520 nm, and **c** 650 nm laser irradiations. **d** Photoresponsivity versus laser power for the 450 nm, **e** 520 nm, and **f** 650 nm laser irradiations

Figure 6a and c show the I-V characteristic curves of the photodetector fabricated using the photolithographic electrode process under laser wavelengths of 450 nm, 520 nm, and 650 nm, respectively, with varying laser powers. The voltage and current exhibit a nearly linear relationship, indicating that the interface between the photolithography electrode and the MoS_2_ nanosheet is still predominantly ohmic, similar to the previously mentioned surface sulfur vacancy defects in the MoS_2_ nanosheets. Figure 6d and f depict the photoresponsivity of the photodetector at varying laser powers and biases under the same wavelengths. Under the low power of the 450 nm laser at 5 V, the maximum photoresponsivity is approximately 3.62 mA W^−1^; for the 520 nm laser at 5 V, it is about 2.45 mA W^−1^; and for the 650 nm laser at 5 V, it is around 1.51 mA W^−1^. Compared to the electrode transfer process, the photolithography electrode process photodetector, with its narrower channel, can more effectively collect the carriers, resulting in a significantly higher photoresponsivity at the same laser power.

The relatively high dark current is an important factor affecting the sensitivity of the photodetector. Assuming that the dominant noise source is the shot noise associated with the dark current, the shot-noise-limited noise equivalent power (NEP) and specific detectivity (D*) can be estimated as (2*eI*_*d*_)^1/2^/*R* and *R*/(2*eJ*_*d*_)^1/2^, respectively, where *R* is the photoresponsivity, *I*_*d*_ is the dark current, *J*_*d*_ is the dark current density calculated from the measured dark current and the effective electrode contact area, and *e* is the elementary charge [30–32]. Based on an effective electrode contact area of approximately 0.325 mm^2^, a dark current of approximately 20 nA at 5 V bias, and a maximum photoresponsivity of 3.62 mA/W, the dark current density, shot-noise-limited NEP, and D* are estimated to be approximately ~ 6 µA/cm^2^, ~ 2 × 10^–11^ W/Hz^1/2^, and ~ 2.6 × 10^9^ Jones, respectively. These values indicate that although the proposed electrode transfer process successfully preserves the structural integrity of the MoS_2_ nanosheets, the relatively high dark current still limits the detectivity of the device. Future work will focus on optimizing the electrode material to form stronger Schottky contacts and employing higher-quality single-crystal or epitaxial MoS_2_ to suppress leakage current and further improve detectivity.

**Fig. 7:**
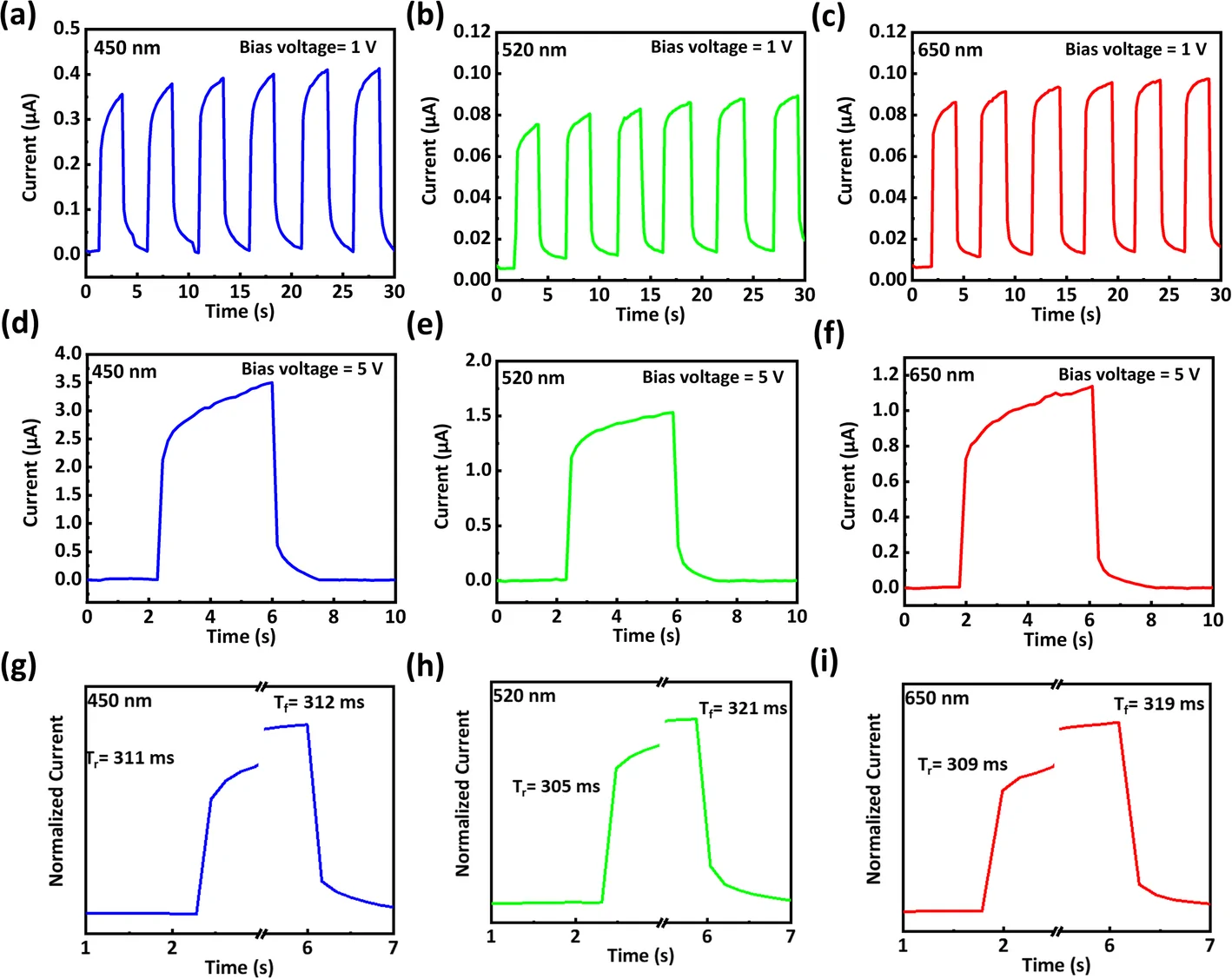
I-t curves of the photodetector with photolithographic electrode process under 1 V bias and **a** 450 nm (multi-cycle), **b** 520 nm (multi-cycle), **c** 650 nm (multi-cycle), **d** 450 nm (single-cycle), **e** 520 nm (single-cycle), **f** 650 nm laser (single-cycle), **g** 450 nm (single-cycle switching current ratio), **h** 520 nm (single-cycle switching current ratio), and **i** 650 nm (single-cycle switching current ratio) laser irradiations

Figure 7a and c present the I-t curves of the photodetector under the three laser wavelengths at a constant bias of 1 V, demonstrating stable light and dark current performance over multiple cycles within a 30-second interval. Figure 7d and f show the I-t curves for single switches. The results indicate that the switching current ratio for the photodetector at a 5 V bias is approximately 10 for the 450 nm laser, about 3.6 for the 520 nm laser, and approximately 7.6 for the 650 nm laser. Figure 7g and i illustrate the response times of the photodetector under 5 V for the 450 nm, 520 nm, and 650 nm lasers (defined by the interval between 10 and 90% of the photocurrent). Regardless of the laser wavelength, the rise time of the photodetector is approximately 308 ms, and the fall time is about 317 ms.

### MoS_2_ nanosheet FET

Figure S19a presents the I_d_-V_g_ logarithmic curves of MoS_2_ nanosheet FETs (channel width/length = 10 μm / 3 μm) under atmospheric conditions at drain voltages of 1 V, 1.5 V, and 2 V. As the gate voltage increases, the drain current also rises, indicating that the MoS_2_ nanosheet behaves as an N-type semiconductor material, with the resulting FET exhibiting NMOS characteristics and an N-type channel primarily facilitated by electron transport [14, 33]. The switching current ratios at the three drain voltages are approximately 10^4^, 5 × 10^3^, and 10^3^, respectively. Based on the measurements, the drain current is converted into a square root versus gate voltage characteristic curve, and the trendline slope in the saturation region is obtained. The electron mobility under different drain voltages is then calculated according to Supplementary material [34]. Figure S19b shows the square root of the drain current versus gate voltage characteristic curve for the MoS_2_ nanosheet FET at a drain voltage of 2 V during forward and reverse sweeps, yielding electron mobilities of 0.014 cm^2^ V^−1^ s^−1^ and 0.17 cm^2^ V^−1^ s^−1^, respectively. Furthermore, it is observed that the MoS_2_ nanosheet FET exhibits different electron mobilities during forward and reverse sweeps at the three drain voltages, with the forward sweep mobility being consistently lower than that during the reverse sweep. This phenomenon is attributed to the influence of moisture and gases in the atmosphere on the defect states in the MoS_2_ nanosheet, which can change under forward and reverse gate voltage sweeps, consequently affecting electron transport.

**Fig. 8:**
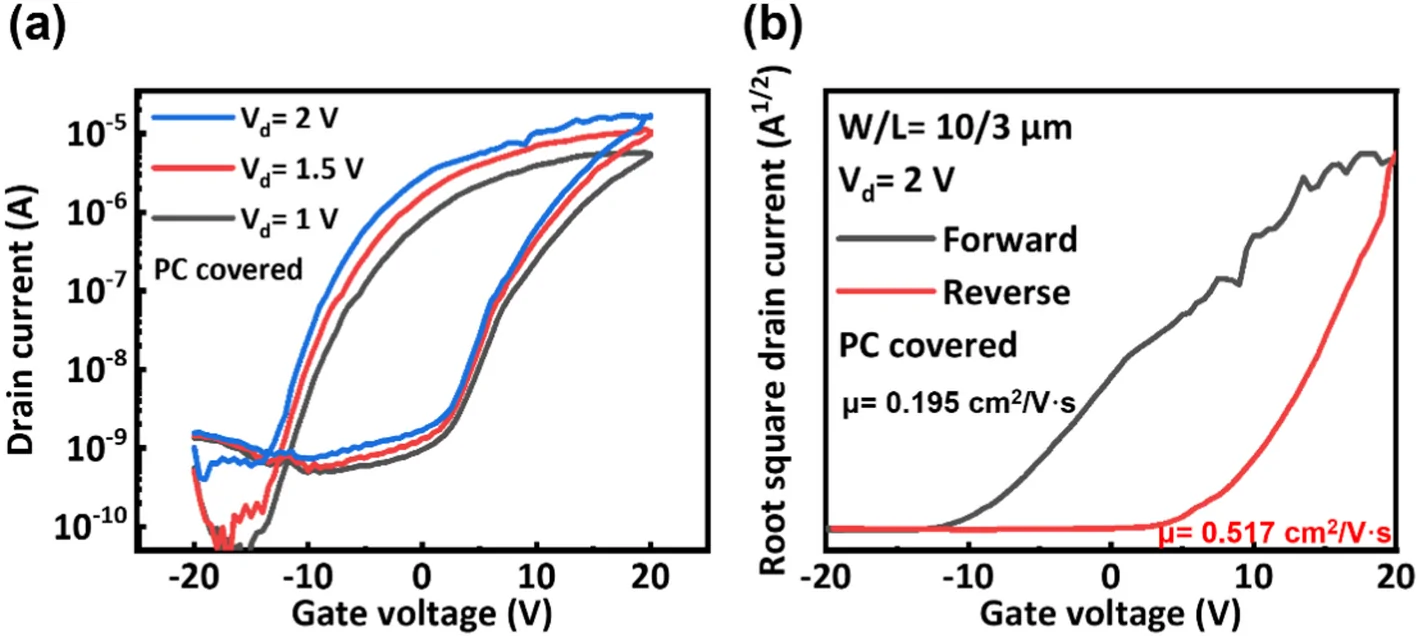
PC-covered MoS_2_ nanosheet FET in atmospheric conditions. **a** I_d_-V_g_ logarithmic characteristic curves at different drain voltages. **b** Electron mobility during forward and reverse sweeps at gate voltage = 2 V

To mitigate the impact of atmospheric moisture and gases on the measurement results, Fig. 8a shows the logarithmic curves of drain current versus gate voltage for PC-covered MoS_2_ nanosheet FETs under ambient conditions (channel width/channel length = 10 μm/3 μm) at drain voltages of 1 V, 1.5 V, and 2 V. These curves demonstrate improved noise performance. The on/off current ratios under the three drain voltages are approximately 10^4^, 5 × 10^3^, and 10^3^, with no significant differences observed. Based on the measurement results, the transfer characteristics are converted into the square root of drain current versus gate voltage curves, and the trend line slope in the saturation region is calculated. Figure 8b presents the square root of drain current versus gate voltage characteristics under forward and reverse sweeps at a drain voltage of 2 V. The calculated electron mobilities are 0.195 cm^2^ V^−1^ s^−1^ and 0.517 cm^2^ V^−1^ s^−1^, respectively. Compared to MoS_2_ nanosheet FETs without PC coverage, the PC-covered MoS_2_ nanosheet FET exhibits closer slopes of the trend lines in the saturation region for forward and reverse sweeps and significantly improved electron mobility. This indicates that PC coverage effectively isolates the MoS_2_ nanosheet from the influence of atmospheric moisture and gases. Figure S20 illustrates the drain current versus drain voltage characteristics of PC-covered MoS_2_ nanosheet FETs at various gate voltages. The increase in current with increasing gate voltage and the positive threshold voltage confirm that the device operates as an n-type enhancement-mode FET. To evaluate whether the electrical characteristics of the electrodes were affected during the transfer process, we have also fabricated FET devices by directly transferring MoS_2_ onto as-deposited electrodes on SiO_2_/Si substrates. For these bottom-gate, bottom-contact FET devices, all samples exhibited mobility values in the range of 0.1–0.5 cm^2^/V s, indicating that the electrical characteristics of the electrodes remain comparable after the transfer process.

Transferred metal electrodes in MoS_2_ nanosheet FETs provide significant advantages by minimizing damage to the MoS_2_ structure during fabrication, preserving its intrinsic properties, and maintaining stable device performance. Compared to conventional deposition methods, transfer electrodes ensure a cleaner interface, contributing to improved electron transport and reduced defect-induced variability. When combined with PC coverage, the devices exhibit even more excellent stability and enhanced performance under ambient conditions. PC-covered MoS_2_ nanosheet FETs with transfer metal electrodes show superior electron mobility (0.195 cm^2^ V^−1^ s^−1^ for forward sweeps and 0.517 cm^2^ V^−1^ s^−1^ for reverse sweeps), reduced discrepancies between forward and reverse sweep mobilities and adequate isolation from atmospheric moisture and gases. Additionally, these devices operate as n-type enhancement-mode FETs with stable on/off current ratios and positive threshold voltages, highlighting the benefits of combining transfer electrodes and PC coverage for reliable and high-performance MoS_2_-based FETs.

To further highlight the significance of this work, Table S1 has been used to compare the proposed PC-assisted transfer process with previously reported electrode transfer methods. Compared with previously reported methods, many transfer approaches require substrate pretreatment, sacrificial layers, multiple supporting polymers, or relatively complicated transfer procedures. In contrast, the proposed method employs standard Si or SiO_2_/Si substrates and utilizes a single PC support layer for electrode transfer without additional substrate pretreatment or chemical etching. Metal electrodes are first deposited onto the substrate, coated with PC, released using DCM, and subsequently transferred onto the target TMD layer through a simplified four-step process. This simplified process reduces fabrication complexity and may lower process cost. Furthermore, unlike conventional approaches that transfer TMD materials themselves, the present work transfers pre-patterned metal electrodes, thereby avoiding direct high-energy metal deposition on ultrathin MoS_2_ nanosheets and reducing potential structural damage. The successful transfer of large-area electrode arrays indicates the capability for simultaneous transfer of multiple patterned electrodes and suggests potential scalability for large-area device fabrication. We believe that the comparison table together with the additional discussion more clearly highlights the practical significance and unique characteristics of the proposed PC-assisted transfer strategy. It should be noted that the compared studies involve different material systems, device architectures, channel dimensions, fabrication processes, and measurement conditions. Therefore, these performance metrics are presented only as representative values to illustrate the capabilities of each transfer strategy rather than for direct quantitative comparison among different studies.

## Conclusions

This study successfully employed PC for the shadow mask process and photolithography to transfer metal electrodes. During the transfer process, optical microscopy and AFM were used to confirm that the metal electrodes were not severely affected, leading to the successful fabrication of top-contact single-layer MoS_2_ nanosheet photodetectors and FETs. Both optical microscopy and Raman and PL spectroscopy confirmed that the MoS_2_ nanosheets remained intact during the metal transfer process. Compared to traditional deposition techniques, this method significantly reduces high-energy impacts on semiconductor materials, thereby enhancing the performance of semiconductor devices. Among the devices fabricated, the photodetector using the shadow mask process (channel length = 20 μm) exhibited a maximum photoresponsivity of approximately 0.73 µA W^− 1^ under a 0.08 mW 450 nm laser and a 10 V bias voltage, with a switching current ratio of about 100. In contrast, the photodetector made via photolithography (channel length = 8 μm) demonstrated a maximum photoresponsivity of approximately 3.62 mA W^− 1^ at a 0.08 mW 450 nm laser and a 5 V bias, with a switching current ratio of around 10. The field-effect transistor fabricated by photolithography (channel width/length = 10 μm / 3 μm) exhibited the highest electron mobility of 0.17 cm^2^ V^− 1^ s^− 1^ during reverse sweep at a drain voltage of 2 V under atmospheric conditions, achieving a switching current ratio of about 10^4^. To mitigate the effects of atmospheric gases and moisture on measurements, the PC-covered field-effect transistor under the same conditions showed an increased maximum electron mobility of 0.517 cm^2^ V^− 1^ s^− 1^ with a similar switching current ratio of 10^4^. The I-V characteristic curve confirmed its operation as an n-type channel enhancement-mode field-effect transistor. This study offers a novel metal electrode transfer process for semiconductor device fabrication, presenting more straightforward, faster, and cost-effective advantages than existing methods. This work is anticipated to contribute to the future application of transition metal dichalcogenide nanosheets in semiconductor devices and integrated circuits.

## Supplementary Information

Below is the link to the electronic supplementary material.

Supplementary Material 1.

## Data Availability

All data that support the findings of this study are included within the article and its Supplementary Information. The datasets and raw data generated and/or analyzed during the current study are available from the corresponding author, Meng-Lin Tsai (mltsai@mail.ntust.edu.tw), upon reasonable request.
