# Supplementary material for: Metal electrodes transfer using polycarbonate for the fabrication of molybdenum disulfide semiconductor devices
Source: Discov Nano. 2026 Aug 26;21(1):416. doi: 10.1186/s11671-026-04877-z (PMC13518768; doi:10.1186/s11671-026-04877-z)
Supplement: Supplementary file 1 [file 11671_2026_4877_MOESM1_ESM.docx]

**Supplementary Information**

**Metal Electrodes Transfer Using Polycarbonate for the Fabrication of Molybdenum Disulfide Semiconductor Devices**

Chih-Hao Chiang, Ruo-Yao Wang, Jing-Ting Chou, Yun-Ping Chiu, Che-Wei Chu, Yueh-Wei Chen, Guan-Ting Chen, Zi-Rui Su, Chi Chen, Chen-Fang Kang^*^, Jhong-Ren Huang^*^, and Meng-Lin Tsai^*^

**Device Applications, Measurements, and Parameter Calculations of Transition Metal Dichalcogenides**

**Photoresponsivity**

Photoresponsivity refers to the response of a photonic device to incident light. It is typically expressed as the output current or voltage generated per watt of incident optical power. It is one of the key parameters for evaluating the performance of photonic devices. Photo-responsivity is commonly denoted as R, and its calculation formula is:

$$R=\frac{I_{light}-I_{dark}}{P_{in}}=\frac{I_{photo}}{P_{in}}$$

Equation S1

Where:

I_light_: Bright current (current under illumination),

I_dark_: Dark current (current in the absence of illumination),

I_photo_: Photocurrent, calculated as I_photo_ = I_light_ - I_dark_

P_in_: Incident optical power (in watts).

The unit of photo-responsivity (R) is amperes per watt (A/W) when the output signal is current, or volts per watt (V/W) when the output signal is voltage.A higher photo-responsivity indicates that the photonic device has a stronger ability to sense incident light and can generate a larger output signal, making it more sensitive and effective for optical signal detection.

**On-Off Current Ratio**

The on-off current ratio is defined as the ratio of the output current under illumination I_light_ to the output current in the absence of illumination I_dark_ at a fixed bias voltage.

The formula is expressed as:

$$On-off ratio=\frac{I_{light}}{I_{dark}}$$

Equation S2

A higher on-off current ratio indicates stronger sensitivity of the optoelectronic device to incident light, demonstrating superior device performance.

**Photo-Response Time**

The photo-response time describes the response speed of a photodetector to the turning on and off of the incident light source. A shorter photo-response time indicates better photo-response performance of the device.

The photo-response time consists of two components:

**Rise Time (t_r_)**: The time required for the output current to increase from 10% to 90% of its maximum value when the light source is turned on.

**Fall Time or Decay Time (t_f_ or t_d_)**: The time required for the output current to decrease from 90% to 10% of its maximum value when the light source is turned off.

These metrics are commonly used to define the dynamic characteristics of photodetectors, providing insight into their ability to respond quickly to changes in illumination.

**On-Off Current Ratio**

The on-off current ratio is defined as the ratio of the output current of the device in the "on" state to that in the "off" state at a fixed drain voltage. The formula is expressed as:

$$On-off ratio=\frac{I_{on}}{I_{off}}$$

Equation S3

Where I_on_ and I_off_ represent the output current in the on and off states of the channel, respectively. A high on-off current ratio enables the device to distinguish on and off signals at lower gate voltages, thereby reducing device heating.

**Carrier Mobility**

Carrier mobility refers to the relationship between carrier velocity and the electric field in a semiconductor material under the presence of an electric field. It describes the ability of carriers to move in response to an external electric field, where the carriers can be electrons or holes, depending on whether the semiconductor is n-type (electrons) or p-type (holes). Carrier mobility is typically denoted by μ and is expressed in units of cm^2^/V·s, representing the distance a carrier drifts in the material under an electric field generated by a voltage of 1 volt. The carrier mobility is influenced by the material's structure and intrinsic properties. High mobility is critical for the performance of electronic devices, as it impacts key parameters such as transistor speed and electrical conductivity.

In MOSFETs, when the gate voltage is sufficient to open the channel, the corresponding voltage is referred to as the threshold voltage (V_th_). At this point, the drain current–drain voltage (I_D_-V_D_) characteristic curve transitions from a linear-parabolic region to saturation, as shown in Fig S19. The calculation of carrier mobility differs between these two regions, but the mobility in the saturation region is generally used as a reference. Carrier mobility is influenced by factors such as the channel length and width, the dielectric constant (ε) of the gate oxide, and its thickness. The mobility can be derived using the ideal current–voltage relationship in the saturation region, expressed as:

$$I_{d}=\frac{W\mu\varepsilon_{ox}}{2Lt_{ox}}\left( V_{g}-V_{th} \right)^{2}$$

Equation S4

Where:

I_d_: Drain current,

W: Channel width,

L: Channel length,

ε_ox_: Dielectric constant of the gate oxide,

t_ox_: Thickness of the gate oxide,

V_G_: Gate voltage.

The ratio of ε_ox_ to t_ox_ is equivalent to the unit capacitance of the gate oxide C_ox_. Therefore, Equation 4 can be rewritten as:

$$I_{d}=\frac{W\mu C_{ox}}{2L}\left( V_{g}-V_{th} \right)^{2}$$

Equation S5

Taking the square root of Equation (5) yields:

$$\sqrt{I_{d}}=\sqrt{\frac{W\mu C_{ox}}{2L}}\left( V_{g}-V_{th} \right)$$

Equation S6

The measured drain current–gate voltage (I_D_-V_G_) characteristic curve is transformed into a square root of drain current–gate voltage characteristic curve. The red line represents the trend line of the $\sqrt{I_{d}}$- V_G_ characteristic curve in the saturation region. The intersection of this trend line with the X-axis corresponds to V_th_ (threshold voltage). At this point, Equation (6) represents the linear equation of the trend line, where the coefficient equals the slope of the line:

$$S=\sqrt{\frac{W\mu C_{ox}}{2L}}$$

Equation S7

Rearranging and simplifying Equation (7) yields:

$$\mu=\frac{2LS^{2}}{WC_{ox}}$$

Equation S8

**Fig S1.** Schematic illustration of the chemical vapor deposition (CVD) process for MoS_2_ nanosheet growth.

**Fig S2.** Heating profile of the chemical vapor deposition (CVD) process for MoS_2_ nanosheet growth.

**Fig S3.** Photographic and optical microscopic images of as-grown MoS_2_ nanosheets on sapphire substrates.

**Fig S4.** Schematic illustration of the preparation of polycarbonate transfer solution.

**Fig S5.** Schematic illustration of the fabrication process of shadow mask electrodes.

**Fig S6.** Schematic illustration of the photolithography electrode fabrication process.

**Fig S7.** Schematic illustration of the photodetector structure.

**Fig S8.** Schematic illustration of the electrode transfer process for the photodetector.

**Fig S9.** Schematic illustration of the structure of the field-effect transistor.

**Fig S10.** Optical microscopic images of (a) as-grown MoS_2_ nanosheets on the sapphire substrate and (b) transferred MoS_2_ nanosheets on the SiO_2_/Si substrate.

**Fig S11.** Tauc plot of MoS_2_ nanosheets.

**Fig S12.** (a) OM and photographic images of the electrodes covered MoS_2_ before and after electrode removal for the electrode transfer process. (b) OM and photographic images of the electrodes covered MoS_2_ before and after electrode removal for the electrode deposition process.

**Fig S13.** (a) Raman and (b) PL spectra of MoS_2_ nanosheets before the electrode transfer, after the electrode transfer process, and after the electrode deposition process.

**Fig S14.** (a) AFM image and (b) height profile of the electrode before transfer. (c) AFM image and (d) AFM height profile curve of the electrode after transfer.

**Fig S15.** (a) Low magnification OM and photographic images of the electrodes before transfer. (b) High magnification OM image of the electrode channel before transfer. (c) OM image and actual photo of the original substrate after electrode transfer. (d) OM image and actual photo of the electrode on the PC film. (e) OM image of the electrode channel on the PC film. (f) OM and photographic images of the transferred electrode on the MoS_2_ nanosheets. (g) OM image of the electrode channel after transfer.

**Fig S16.** (a) I-V characteristic curves of the photodetector with shadow mask electrode process under 450 nm, (b) 520 nm, and (c) 650 nm laser with different powers. (d) Photoresponsivity versus laser power for 450 nm, (e) 520 nm, (f) and 650 nm laser at different bias voltages.

**Fig S17.** I-t curves of the photodetector with shadow mask electrode process under 10 V bias and (a) 450 nm (multi-cycle), (b) 520 nm (multi-cycle), (c) 650 nm (multi-cycle), (d) 450 nm (single-cycle), (e) 520 nm (single-cycle), and (f) 650 nm (single-cycle) laser irradiation.

**Fig S18.** Response times of the photodetector with shadow mask electrode process under 10 V bias. (a) Rise and (b) fall times under 450 nm laser. (c) Rise and (d) fall times under 520 nm laser. (e) Rise and (f) fall times under 650 nm laser.

**Fig S19.** MoS_2_ nanosheet FET in atmospheric conditions. (a) I_d_-V_g_ logarithmic characteristic curves at different drain voltages. (b) Electron mobility during forward and reverse sweeps at gate voltage = 2 V.

**Fig S20.** I_d_-V_d_ characteristic curves of PC-covered MoS_2_ nanosheet FET under different gate voltages in atmospheric conditions.

**Table R1.** Comparison of different metal electrode transfer methods.

| **Device** | **Pre-treatment** | **Post-treatment** | **Pre-deposited**  **substrate** | **Polymer** | **Polymer removal** | **Processing steps** | **Mobility**  **(cm^2^/V·s)** | **Responsivity** | **Detectivity**  **(Jones)** | **Ref.** |
| --- | --- | --- | --- | --- | --- | --- | --- | --- | --- | --- |
| MoS_2_ FET/PD | HDMS functionalized | - | Si | PDMS stamp  PMMA | E-beam lithography | 7 | 260 | 16.6 mA/W | - | [S1] |
| Organic TFT | F-SAM treatment | - | Glass | Adhesive tape  PMMA  PVA | DI water | 8 | 9.5 | - | - | [S2] |
| MoS_2_ FET | Graphene deposition | - | Ge | PDMS stamp  PVA | DI water | 7 | 5.9 | - | - | [S3] |
| InSe PD | - | KOH/ NaOH etching | SiO_2_/Si | Heat release tape  PMMA | Acetone | 7 | 0.036 | 6.95 × 10^5^ A/W | 1.7 × 10^12^ | [S4] |
| **MoS_2_ FET/PD** | **-** | **-** | **Si**  **SiO_2_/Si** | **PC** | **DCM** | **4** | **0.52** | **3.62 mA/W** | **2.6 × 10^9^** | **This study** |

**References**

(S1) Liu Y, Guo J, Zhu E, Liao L, Lee SJ, Ding M, Shakir I, Gambin V, Huang Y, Duan X. Approaching the Schottky–Mott limit in van der Waals metal–semiconductor junctions. Nature. 2018;557:696-700. <https://doi.org/10.1038/s41586-018-0129-8>.
(S2) Makita T, Yamamura A, Tsurumi J, Kumagai S, Kurosawa T, Okamoto T, Sasaki M, Watanabe S, Takeya J. Damage-free metal electrode transfer to monolayer organic single crystalline thin films. Sci Rep. 2020;10:4702. <https://doi.org/10.1038/s41598-020-61536-8>.
(S3) Liu G, Tian Z, Yang Z, Xue Z, Zhang M, Hu X, Wang Y, Yang Y, Chu PK, Mei Y, Liao L, Hu W, Di Z. Graphene-assisted metal transfer printing for wafer-scale integration of metal electrodes and two-dimensional materials. Nat Electron. 2022;5:275-80. <https://doi.org/10.1038/s41928-022-00764-4>.
(S4) Jin K, Li T, Cai H, Li M, Pan N, Wang X. Transfer printing of metal electrodes for high performance InSe photodetectors. Opt Commun. 2019;436:47-51. <https://doi.org/10.1016/j.optcom.2018.11.076>.
